# Is 12 months enough to reach function after athletes’ ACL reconstruction: a prospective longitudinal study

**DOI:** 10.1016/j.clinsp.2022.100092

**Published:** 2022-09-07

**Authors:** Ellen Cristina Rodrigues Felix, Angelica Castilho Alonso, Guilherme Carlos Brech, Tiago Lazzaretti Fernandes, Adriano Marques de Almeida, Natália Mariana Silva Luna, Jose Maria Soares-Junior, Edmund Chada Baracat, Arnaldo José Hernandez, Júlia Maria D'Andrea Greve

**Affiliations:** a Laboratory Study of Movement, Instituto de Ortopedia e Traumatologia do Hospital das Clínicas (IOT-HC) da Faculdade de Medicina da Universidade de São Paulo (FMUSP), São Paulo, SP, Brazil; b Graduate Program in Aging Sciences from the Universidade São Judas Tadeu (USJT), Santos, SP, Brazil; c Sports Medicine Division, Institute of Orthopaedics and Traumatology, Hospital das Clínicas, Faculdade de Medicina, Universidade de São Paulo; FIFA Medical Centre of Excellence, São Paulo, SP, Brazil; d Disciplina de Ginecologia, Departamento de Obstetrícia e Ginecologia, Hospital das Clínicas da Faculdade de Medicina da Universidade de São Paulo (FMUSP), São Paulo, SP, Brazil

**Keywords:** Postural balance, Isokinetic evaluation, Return to sport, Anterior cruciate ligament injuries

## Abstract

**Context:**

Anterior Cruciate Ligament (ACL) injury is disabling in several sports because it causes knee instability and functional deficit. Usually, surgical treatments produce the best functional outcomes, however, sometimes they are not always able to fully restore stability and function.

**Objective:**

The objective of this study was to evaluate postural balance, muscle strength, and functional performance of young athletes with an ACL injury before and after ACL reconstruction.

**Design:**

This was a longitudinal observational prospective study.

**Method:**

74 athletes, 60 men, and 14 women, aged between 16 and 45, divided into two groups: the Group-Lesion of ACL with 34 athletes (24.1 years) and the Group-Control with 40 athletes without ACL lesion (27.7 years old). All volunteers performed posturography, isokinetic dynamometry, and the Hop-Test. The ACL-Group was evaluated before and 12 months after the reconstruction and the control group was evaluated once.

**Results:**

The Postoperative ACL Group presented greater limb symmetry, 0.96 (± 0.12), than the preoperative ACL Group, 0.87 (± 0.17), *p* < 0.01 in the Hop-Test. In the posturography, the displacement area was smaller in the postoperative ACL Group, 19.85 (± 5.74), compared to the preoperative ACL Group, 24.20 (± 8.97), *p <* 0.01. In isokinetic dynamometry the torque peak was greater in the postoperative ACL Group, 0.91 (± 0.14), than in the preoperative ACL Group, 0.74 (± 0.15), *p <* 0.01.

**Conclusion:**

The functional outcomes increased in ACL reconstruction athletes after 12 months, but not at the same level as in the Control Group. The result indicates an incomplete functional recovery, adaptive changes in postural control after injury, reconstruction, and return to sport.

## Highlights

• Anterior cruciate ligament injury is disabling in several sports.
• nterior cruciate ligament injury causes knee instability and functional deficit.
• Usually, surgical treatments produce best functional outcomes.

## Introduction

Anterior Cruciate Ligament (ACL) injury leads to knee instability, functional deficit, and other joint structure lesions such as meniscus.^1^ It is very disabling for various sporting activities, and it increases the risk for osteoarthritis.^2^

After an ACL injury, the mechanoreceptors fail to send information for neuromuscular responses to the maintenance of balance and joint function, presenting some postural control system instability and muscle strength deficits.^3^ Besides, quadriceps and hamstrings muscles are also influenced by motor control, the consequence is a decrease in quadriceps activation and greater flexor muscle activity of the knee.^4^

Ligament reconstruction restores anteroposterior stability but does not completely restore rotational stability.^5^ Even after surgical intervention, studies show that proprioceptive deficit remains.^3^^,^^6^

Some authors^7^^,^^8^ reported functional deficits in athletes after returning to sports. Only 33% of ACL reconstructed patients with hamstring graft and 41% with patellar graft have returned to their pre-injury sport condition,^9^ and it increases the risk of reinjury.^10^

The most common measure of functional recovery during the rehabilitation program are clinical, functional assessments evaluating muscle strength, postural control, and postural balance.^11^ The most commonly used clinical assessments are:^12^ “Hop-Tests” – dynamic functional tests that perform tasks that measure performance;^13^ Isokinetic dynamometry torque and muscle function (strength);^14^ Posturography ‒ force platforms for postural balance evaluation and postural control.^6^ Functional tests are easy to perform and useful, “Hop-Tests”^13^ quantitative measures such as isokinetic dynamometry, and static and dynamic posturography^5^^,^^15^ are more accurate to measure the patient's functional improvement, allowing a return to sports with more security.^16^

The controversy of the present study is that decreased muscle strength, dynamic knee stability, and functional performance after ACL after 12 months postoperatively could be associated with incomplete recovery of the knee stabilizing mechanism.

Therefore, the aim of this study is to assess postural balance, muscle strength, and functional performance based on measures made upon a group of athletes with an ACL injury before and 12 months after surgery and matched control subjects.

## Methods

### Experimental design, local and ethics

A 12-month longitudinal observational prospective study was approved by the Local Ethics Committee of the Universidade de São Paulo (number 652.361) and all of the subjects signed the informed consent.

The study was performed at the Motion Study Laboratory of the Institute of Orthopedics and Traumatology, Hospital das Clínicas, University of São Paulo School of Medicine.

### Subjects

The subjects were recruited into the study by a group of Sports Medicine from the hospital. The sample was composed of 74 athletes from different sports activities who were included in this study. The inclusion criteria for this study were: (1) Recreational or professional athletes; (2) Age between 15 and 45 years old; (3) Sports practice of at least 12 months; (3) Five or above in physical activity Tegner activity scale; (4) No history of medical problems that limited activities within the 6 weeks before testing; (5) Primary injury in ACL was accepted only for the ACL Injury Group. The exclusion criteria were: (1) History of previous knee injuries or leg surgeries; (2) Associate lesions such as meniscus, cartilage, or other ligament injuries; (3) Knee valgus and varus alignments; (4) For the Control Group, knee instabilities.

### Allocation

The athletes were in two groups: the ACL injury Group (27 men and 7 women) and the Control Group (athletes without the ACL injury, 33 men and 7 women). The ACL Group was a consecutive group of injured athletes that underwent ACL reconstruction surgery by two expert surgeons (from the Sports Medicine Team), and the time between the ACL injury to the first evaluation are more than three months apart in order to avoid the inflammatory period and a greater functional disability, a restricted range of motion or swelling. The Orthopedists from the team assessed the volunteers by magnetic resonance imaging, clinical maneuvers, and history of recurrent knee instability. The Control Group was selected in agreement with the ACL Group's age, gender, and level of physical activities by the Tegner activity scale. The subjects of the ACL group were assessed at baseline (preoperative) and 12 months after surgery. The primary outcomes were the postural balance measures, as assessed by the force plate. Secondary outcomes included physical function tests and strength assessment.

Follow-up was also performed by orthopedists at the hospital 3 weeks, and 4, 6, 9 and 12 months following surgery. Patients allocated to the ACL group were given detailed instructions about the rehabilitation program (rehabilitation protocol) and they could choose the rehabilitation center. The rehabilitation progress was checked during the follow-up visits.

### Postural balance

**Fig. 1:**
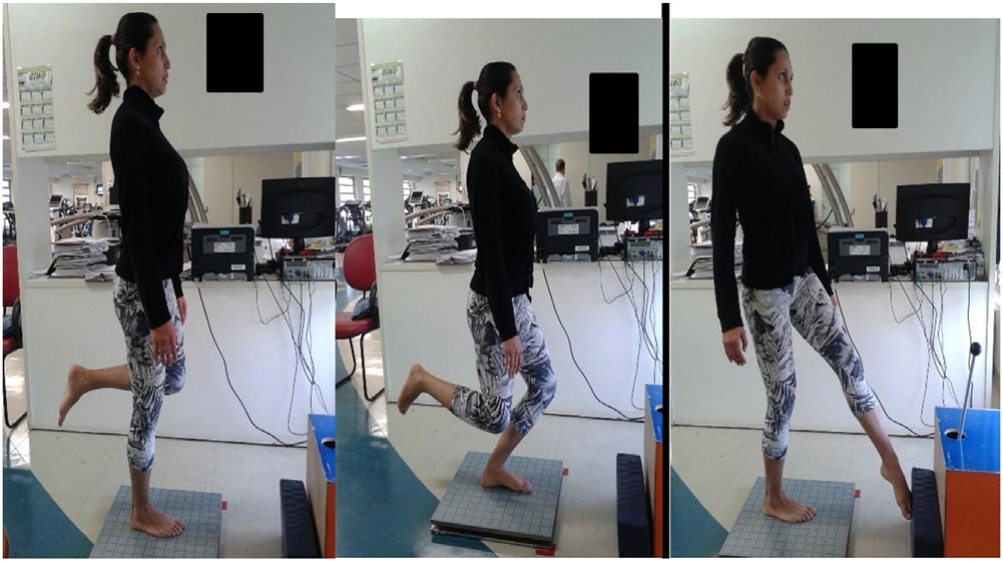
Posturography ‒ A e B: squat (knee flexion); C, kick movement.

The postural balance assessment (posturography) was performed on a portable force platform (AccuSway Plus, AMTI®, MA, USA). For data acquisition, the force platform was connected to a signal-amplifying interface box (PJB-101) that was linked to a computer by means of an RS-232 cable. The data was gathered and stored using Balance Clinic® software, configured to a frequency of 100 Hz with a fourth-order Butterworth filter and a cutoff frequency of 10 Hz. All subjects underwent the test with standardized positioning. Two tests were performed (Fig. 1), all with single-leg support: knee flexion up to 45° (as squat associated with straight trunk position) (Squat Test – Fig. 1B), and rotation of the trunk and hip (simulating a kick with the outside edge of the foot) (Kick Test – Fig. 1C).^17^ The last test was performed through internal rotation of the trunk on the supporting limb, with movement constantly repeated during the test period. The movement speed was established according to the strategy adopted by each volunteer. Each test was performed three times. Knee flexion movement (squat) and kick simulation tests lasted 10 s. There was an interval of 30 s between each test. The arithmetic means of the results were calculated from the three tests conducted under each condition and were processed using the Balance Clinic® software. The parameters used to measure the subject's stability were the average of the Center of Pressure (COP) displacement in the anteroposterior direction (cm); the average of the COP displacement in the mediolateral direction (cm); the velocity of displacement oscillation (cm/sec); and the area (95% of the area formed by the ellipse of the trajectory from the COP).^17^

### Functions mobility assessment

The Single Leg Hop Test for a distance consists of a horizontal jump to the farthest possible distance using only one leg and keeping the hands behind the body. Three jumps were performed with each leg, with an interval of one minute between them, and the arithmetic average of the distances of each jump was used.^18^ The single hop was considered successful if the landing was stable. To be considered a valid trial, the landing must be on one limb, under complete control of the patient's stability. If the patient landed with an early touchdown of the contralateral limb, had a loss of balance, or had additional hops after landing, the hop would be repeated. Patients were instructed to begin with the uninjured leg, with their lead toe behind a marked starting line. The hop distance was measured to the nearest centimeter from the starting line to the patient's heel with a standard tape measure.

### Muscle strength assessments

Isokinetic dynamometry was performed using the Biodex® Multi-joint System 3 (Biodex Medical^TM^, Shirley, NY, USA. The angular velocity used in the isokinetic test was 60⁰/s. The isokinetic dynamometer was calibrated thirty minutes before starting the tests. After a standardized warm-up, the subjects were positioned for a concentric evaluation of extension and flexion movements of the knee joint. They remained seated with the hips at 90° of flexion and were secured to the chair by belts. The test was started with the uninjured limb. The limb was evaluated by positioning the lateral condyle of the femur in alignment with the mechanical axis of the dynamometer. All subjects performed four submaximal repetitions to become familiar with the equipment, followed by a 60-second rest interval, then two series of four maximal repetitions of knee extension and flexion starting with the dominant limb, with a 60-second interval between the series. The values from the second series were used for data analysis regarding the effects of motor learning on clinical isokinetic performance. Constant standardized verbal encouragement was given during the tests in order to promote maximum effort during contractions. The isokinetic variable used was maximum Peak Torque corrected for Body Weight (PTQ/BW). Values were used to calculate Limb Index Symmetry (LSI): operated/non-operated limb.

### Statistical analysis

The sample size calculation was based on a pilot study with 30 subjects (15 with ACL Injury and 15 without an ACL Injury) and the variable used was the COP displacement direction average during the test after the surgery was considered. The average COP displacement for the ACL Group was 7.63 cm with a standard deviation of 1.84, and for the Control Group, it was 6.94 cm with a standard deviation of 1.22. To determine the sample size to compare the two means, the power of the test was set at 80% with a 5% level of significance. To meet these conditions, at least 40 subjects were needed in each group, considering a 10% of loss of follow-up.

The comparison between the means of the quantitative variables related to the demographic data of the participants was carried out through the *U* Mann Whitney test.

For hop-test analysis, isokinetic and dynamic evaluations of the force platform, the Shapiro-Wilk normality test was performed. *U*-Mann-Whitney test and Wilcoxon were used for non-parametric values, all the tests were carried out at an alpha level of 0.05, and the power of the study

Demographic data was calculated using *U*-Mann-Whitney. For hop-test analysis, isokinetic and dynamic evaluations of the force platform, Shapiro-Wilk normality test was performed. *U*-Mann-Whitney test and Wilcoxon were used for non-parametric values, all the tests were carried out at an alpha level of 0.05, and the power of study was equal to 80% (SPSS-9 for Windows). Besides, Cohen's D was used as a measure of effect size.

## Results

The baseline analysis is presented in Table 1.

**Table 1:** Baseline characteristics of patients of both groups.

	ACLG (*n* = 34)		CG (*n =* 40)		
	Mean	SD	Mean	SD	p
Age (years)	25.05	6.82	27.7	8.16	>0.05
Body mass (kg)	72.11	10.19	70.50	15.21	>0.05
Height (m)	1.74	0.69	1.72	0.88	>0.05
BMI (kg/m²)	23.62	2.72	23.68	0.29	>0.05
Tegner activity scale	7.6	1.2	8.0	1.3	>0.05

	F (%)		F (%)		
Gender					
Male	79.4%		82.5%		>0.05
Female	20.6%		17.5%		>0.05
Category					
Professional	35.3%		27.5%		>0.05
Amateur	64.7%		72.5%		>0.05

ACLG, Anterior Cruciate Ligament Group; CG, Control Group; SD, Standard Deviation; F, frequency.

*p-value ≤ 0.05.

There were no statistical differences between the groups regarding the baseline characteristics (Table 1).

The Tegner activity level scale scores were 8 (± 1.3) before and 7.1 (± 1.82) after ACL reconstruction in the ACL group and in the Control group were 7.6 (± 1.2).

Posturography assessment was shown in Table 2.

**Table 2:** Posturography: area (cm^2^) of the center of pressure oscillation during Squat and Kick test.

	ACLG (*n =* 34)				CG (*n =* 40)		
	Preoperative		Postoperative			p	Effect size (d)
	Operated	Non operated	Operated	Non operated			
	Mean (SD)	Mean (SD)	Mean (SD)	Mean (SD)	Mean (SD)		
Kick Test	26.29 (10.92)	31.77 (11.88)^c^^,^^d^	24.38 (8.09)	24.91 (6.39)^d^	23.36 (7.22)^c^	<0.01	0.03‒0.22
Squat Test	24.20 (8.97)^a^	24.05 (10.42)	19.85 (5.74)^a^	20.62 (7.51)	20.08 (6.51)	<0.01	0.57

ACLG, Anterior Cruciate Ligament Group; CG, Control Group; (d) Cohen's D: measure of effect size.

a Significant between preoperative ACL Group and postoperative ACL Group. p ≤ 0.05, Wilcoxon test. ^b^Significant between preoperative ACL Group and Control Group. p ≤ 0.05, Mann Whitney test.

c Significant between postoperative ACL Group and Control Group. p ≤ 0.05, Mann Whitnney test.

d Significant between preoperative ACL Group non operated limb and postoperative ACL Group non operated limb. p ≤ 0.05, Mann Whitnney test.

In the Kick Test, the area of COP oscillation decreased in the postoperative ACL Group only in the non-operated limb compared to the preoperative period, and Control Group (*p <* 0.01; Effect Size = 0.22). In preoperative the ACL group the non-operated limb showed greater oscillation than the Control Group (*p <* 0.01; Effect Size = 0.03).

In the Squat Test, the area of COP was smaller in the ACL Group when postoperative than in preoperative (*p <* 0.01; Effect Size = 0.57).

Single-Leg Hop Test and Isokinetic evaluation are shown in Table 3.

**Table 3:** Single-Leg Hop Test (limb symmetry Index) and Isokinetic dynamometer (knee muscular strength).

	ACLG (*n =* 34)		CG (*n =* 40)		
	Preoperative	Postoperative		p	Effect size (d)
	Mean (SD)	Mean (SD)	Mean (SD)		
Single-Leg Hop Test	0.87 (0.17)^a^^,^^b^	0.96 (0.12)^a^	1.02 (0.95)^b^	<0.01	0.08‒0.61
PT/W					
Quadriceps	0.74 (0.15)^a^^,^^b^	0.91 (0.14)^a^^,^^c^	0.98 (0.11)^b^^,^^c^	<0.01	0.57‒1.87
Hamstring	0.88 (0.18)	0.91 (0.15)	0.94 (0.12)	>0.05	0.05‒0.23

ACLG, Anterior Cruciate Ligament Group; CG, Control Group; PT/W, Peak Torque/Weight; (d) Cohen's D: measure of effect size.

a Significant between preoperative ACL Group and postoperative ACL Group. p ≤ 0.05, Wilcoxon test.

b Significant between preoperative ACL Group and Control Group. p ≤ 0.05, Mann Whitney test.

c Significant between postoperative ACL Group and Control Group. p ≤ 0.05, Mann Whitnney test.

Single-Leg Hop Test showed greater Limb Symmetry (LSI) in the ACL Group when postoperative compared to the preoperative period (*p <* 0.01; Effect Size = 0.61). The Control Group had a greater limb symmetry index than the preoperative ACL Group (*p <* 0.01; Effect Size = 0.21).

Quadriceps showed greater limb symmetry in the ACL when postoperative compared to the preoperative period (*p <* 0.01; Effect Size = 1.19). The Control Group had greater LSI than the ACL Group during the preoperative (*p <* 0.01; Effect Size = 1.87) and postoperative period (*p <* 0.01; Effect Size = 0.59). There was no difference in hamstring strength (limb symmetry index).

## Discussion

The main finding of this study is that, although postural control and muscle strength can suffer adaptive changes after reconstruction in the ACL, nevertheless, there are deficits compared to the control group. Single-Leg Hop Test and the quadriceps strength symmetry limb index increased in the postoperative period in the ACL group compared to preoperative, but not at the same level as the Control Group. Nevertheless, the COP oscillation area decreased in the postoperative period during the Squat Test on operated limbs and during the Kick Test on non-operated limbs, Higher scores in the Tegner Scale were found in the preoperative period showing that preinjury levels were not reached in 12 months following the reconstruction. Volpi et al.^18^ Hohmann et al..^19^ Teitsma et al.,^20^ reported how difficult it is to reach total recovery after the ACL reconstruction. The number of athletes returning to the pre-injury level is growing due to advances in knowledge of ACL injury, reconstruction, and rehabilitation.^21^ Demographic, functional, psychological, and socioeconomic factors may influence the successful safe return to sports.^5^ Some authors^5^^,^^22^ have reported that male gender athletes, age ≤ 24 years old, elite athletes, successfully reconstructed, rehabilitated, and motivated athletes return to sports at the same preinjury level. In the present study, even the athletes that did not return to the supposed same preinjury level showed good recovery in the postoperative period. Higher Tegner scale levels are reached by high-intensity sports that require higher knee stability such as soccer and rugby.

The functions mobility assessment shown in the Single Hop-test (symmetry index), which compares the distances skipped by the two limbs, is an important variable that improves the evaluation of treatment, since it serves as a normalizer of the anthropometric differences. In addition, being a useful measure to compare the two members together.^23^ In the present study, there was an improvement (increase) in the symmetry index in the postoperative period, when compared to the preoperative period. The Control Group showed 100% (index = 1) symmetry, a result that was not fully restored by the postoperative ACL Group, which reached 96% (index = 0.96), but was very close. The preoperative ACL index of symmetry was 88%. The symmetry index ≥ 90% is part of the criteria for returning to the sports but must be evaluated along with the following parameters: muscular strength, postoperative time, and neuromuscular control.

Logerstedt et al.^24^ reported that the symmetry between the limbs in the Single-Leg Hop Test at the end of six months is a predictive factor of good function. Gustavsson et al. (2006)^25^ showed 6% asymmetry in a group of healthy individuals, 21% in the non-operated ACL group, and 20% in the six-month reconstruction group. In the present study, with a 12-month postoperative period, the improvement of the symmetry is observed, at levels that allow the return to sportive activity with safety. This data strongly suggests that a safe return to sports is a multifactorial decision and the time after the surgery is an important aspect but cannot be the only one to be considered.

Another interesting fact was that the non-operated limb showed a better evolution in the postoperative period compared with the operated limb in the Kick Test. The Area of COP oscillation in the preoperative period was greater than in the postoperative period, and when compared to the Control Group. This better response of the non-operated limb can be explained by the integrity of the ACL, because with mechanoreceptors functioning normally, the limb is able to respond better to sensory motor training. In the Squat Test, the operated limbs of the ACL Group decreased their area of COP oscillation in the postoperative period. This lower area of COP displacement may be related to the improvement of the afferents and sensorial responses after the reconstruction. Bryant et al. (2009).^26^ reported changes in muscle activation after reconstruction. Quadriceps activation decreased while the hamstring increased to avoid the anteriorization of the tibia during the movements. It could be expected, also in the Squat Test, some differences in COP oscillation compared to the ACL and Control Group, probably the closed chain movement with higher muscle action of the knee was enough, there wasn't an increased oscillation area, compared to normal individuals.

In two systematic reviews, Negahban et al. (2014),^3^ with non-operated injuries, and Howell et al. (2011),^5^ after reconstruction, the results showed that the injured/operated limbs presented greater displacement of the COP area, besides the higher oscillation velocity when compared with contralateral limb and the Control group. Negahban et al. (2014)^3^ also referred to bilateral but always a greater deficiency in the injured limbs, and Howell et al. (2011)^5^ observed the existence of postural control deficiency two years after reconstruction. The results of the present study are in agreement with those found in the two reviews, although differences in the types of evaluation should be considered. Postural balance is developed and improves physical fitness throughout life that involves several body systems and, therefore, it is hard to evaluate during functional tasks. Postural control is not always compromised in the ACL injury because there is a loss of the ruptured ligament proprioception, but other structures and systems come into action to overcome this failure. This factor may be the basis of the controversial results seen in the assessment of postural balance control in ACL injury: differences between the evaluated patients, the tests used, and the compensatory proprioceptive strategies used. There is a controversy in literature on whether decreasing oscillation of COP is necessary to improve function after ACL reconstruction, maybe the difference in literature is due to adaptive changes to improve function.

Peak torque is the most common variable for measuring muscle strength and it is evaluated by symmetry limb index, which is a criterion to evaluate rehabilitation and return to sports^27^. Lepley et al. (2015)^28^ concluded that muscle strength has more importance on knee function than muscle activation. After 12 months of patellar tendon reconstruction, there was no difference in the hamstring symmetry limb index, in this study. But the quadriceps limb symmetry improved in the postoperative period, but still showed symmetry deficit compared to the Control Group (which means incomplete recovery occurred). This result is not expected, taking into account that the risk of injury is probably due to the effect of muscle training during and after rehabilitation. Some authors, such as Abrams et al.,^13^ Pamukoff et al.^29^ found a peak torque deficit in the extensor muscles and in the flexors postoperatively, the deficit was higher in the quadriceps.

While the extensor muscles (quadriceps) are the most affected in knee injuries, they are also the most important for the postoperative function acquisition and stability in ACL reconstructions^30^. Rehabilitation may influence muscle recovery, mainly because volunteers from the studies are treated with different rehabilitation protocols. Hamstring symmetry did not show improvement, probably because it lost less strength than the quadriceps did. These results differ from the findings of Czaplick et al.^31^ and Konrath et al.^32^ who observed the existence of peak torque deficit in the flexor muscles in flexor tendon reconstructions. These authors evaluated patients 12 to 24 months after surgery, when patients should have returned to sports practice. Many authors use the non-operated limb as a parameter of normality, mainly because many patients are only evaluated during the postoperative period, thus using the limb without injury and/or Control group comparisons. Westin-Barber and Noyes,^33^ under a systematic review, found the index of symmetry among the members as the criterion most cited in the articles, so the authors used the variable in the study. However, the limb without injury can also present a deficit,^34^ as seen in the current study, impairing the evaluation of the patient.

There are some limitations in this study, there was no standardization of rehabilitation in a single center, although they have received detailed instructions about the rehabilitation protocol, and the rehabilitation progress was checked during the follow-up visits; even force platform is considered the gold standard method for balance control, it was difficult to compare this study to others with the same methodology, using the dynamic performance (Squat and Kick Test) in the force platform as used in this present study, even force platform is considered the gold standard method for balance control; and also muscle strength of other parts of the body, knee valgus and kinematics parameters of tests were not evaluated. The authors suggest that future studies should also perform dynamic evaluations, as well as using functional tests, associated with kinematics measures, and thus it would improve the quality of information around parameters related to the COP displacement and functional evaluation in the ACL injury.

One important clinical implication of this study was that even after athletes return to sports they present physical deficits, athletes haven´t returned to the same physical activity level as before the ACL injury.

## Conclusion

The result indicates incomplete adaptive changes in postural control, muscle strength and functional recovery after injury, reconstruction and the return to sport.

### Author's contributions

Ellen Cristina Rodrigues Felix: Investigation and writing-original draft and supporting.

Angelica Castilho Alonso: Supporting investigation and writing-review & editing.

Guilherme Carlos Brech: Supporting investigation and writing-review & editing.

Tiago Lazzaretti Fernandes: Formal analysis and writing-review & editing.

Adriano Marques de Almeida: Formal analysis and writing-review & editing.

Natália Mariana Silva Luna: Investigation and writing-original draft and supporting.

Jose Maria Soares-Junior: Supporting investigation and writing-review & editing.

Edmund Chada Baracat: Supporting investigation and writing-review & editing.

Arnaldo José Hernandez: Supporting investigation and writing-review & editing.

Júlia Maria D'Andrea Greve: Supervisor and writing-review & editing.

## Conflicts of interest

The authors declare no conflicts of interest.
